# Supplementary material for: Memory consolidation in honey bees is enhanced by down-regulation of Down syndrome cell adhesion molecule and changes its alternative splicing
Source: Front Mol Neurosci. 2024 Jan 9;16:1322808. doi: 10.3389/fnmol.2023.1322808 (PMC10803435; doi:10.3389/fnmol.2023.1322808)
Supplement: Supplementary file 1 [file Table_1.docx]

**Supplementary table 1: Oligonucleotides**

**Oligonucleotides**

AM Dscam T7 RNAi 11F GACTCGAGTAATACGACTCACTATAGGGAGAGGATCCTGGAACCCACCGACAAGGCATTC

AM Dscam T7 RNAi 14R GATCTAGATAATACGACTCACTATAGGGAGAGCCACGATTCTGAGGTGATAAGTGGTG

820 bp

AM Dscam GSP 13 RT1 GCCGAGAGTCCTGCGCCGATTCCATTCACAG

AM Dscam 3F1MH AGTTCACAGCCGAGATGTCAAGGTGAGAGCCG

AM Dscam 5R1 GGAAGGCAGTACCAAGTATTTTCCAT

220 bp

AM Dscam 9F2 CGCTAGGGGAACGTTGGAGGTTCAAGTG

AM Dscam 11R2 CGTCGGAGCCTTGAGCGAATGCCTTGTC

380 bp

AM Dscam 5F1A CGTCTAATGGCTTTCGGCTACCAAGGGACGACTCGTCATTAC

AM Dscam 5F1B CCATGAGTGGCTTTCGGCTACCAAGGGACGACTCGTCATTAC

AM Dscam 5F1C GTCATTATGGGCTTTCGGCTACCAAGGGACGACTCGTCATTAC

AM Dscam 7R1A CGTCTAATGCGGGACGAGCCCTCGATGAACTTGTAC

AM Dscam 7R1B CCATGAGTGCGGGACGAGCCCTCGATGAACTTGTAC

AM Dscam 7R1C GTCATTATGGCGGGACGAGCCCTCGATGAACTTGTAC

201 bp, indexes are underlined
