# Supplementary material for: Memory consolidation in honey bees is enhanced by down-regulation of Down syndrome cell adhesion molecule and changes its alternative splicing
Source: Front Mol Neurosci. 2024 Jan 9;16:1322808. doi: 10.3389/fnmol.2023.1322808 (PMC10803435; doi:10.3389/fnmol.2023.1322808)

Memory consolidation in honey bees is enhanced by down-regulation of *Down Syndrome Cell Adhesion Molecule* and changes its alternative splicing

PINAR USTAOGLU[1, 2], DAVID W. J. MCQUARRIE[1, 2], ANTHONY ROCHET[3], THOMAS DIX[1, 2], IRMGARD U. HAUSSMANN[1, 4], ROLAND ARNOLD[2, 5], JEAN-MARC DEVAUD[3, 6] AND MATTHIAS SOLLER[1, 2#]

Suppl. Fig. 1 *Drosophila* anti-Dscam recognizes honey bee Dscam.

A A rabbit polyclonal anti-serum raised against *Drosophila* Dscam recognizes honey bee Dscam on Western blots at expected sizes of 222 and 270 kDa (arrowheads). Extracts of bee brains (lane 2) or head and thorax from *Drosophila* expressing *UAS Dscam* with *daughterlessGAL4* (lanes 3 and 4) were separated on a 8% SDS-gel. Note that the anti-serum recognizes unspecific proteins of 100, 110 and 130 kDa in bees (asterisks). Loading is shown by probing the same blot with anti-tubukin antibodies (bottom). M: Molecular weight marker.

B Western blot of bee brains 48 and 64 h after injection of *Dscam* or *GFP* dsRNA for RNAi knock-down. Note that Dscam levels are reduced after RNAi by injection of Dscam dsRNA (lanes 1 and 3), but not the unspecific proteins of 100, 110 and 130 kDa (asterisks). Loading is shown by probing the same blot with anti-tubulin antibodies (bottom). Molecular weight markers are indicated on the left.

Suppl. Fig. 2 *Apis mellifera Dsacm* exon 10 alternative splicing during bee development and between casts.

A Denaturing polyacrylamide gels showing the splicing pattern of *Dscam* exon 10 isoform variables on top by digestion of a ^{32}P labeled RT-PCR product with a combination of *Rsa*I, *Pvu*II

and *BsaJ*I restriction enzymes in embryos (line1), larval brains (line 2), worker brains (line 3), drone brains (line 4) and queen brains (line 5) and undigested control at the bottom. sk: skipping of all exon 10 variables. Molecular weight markers are indicated on the left.

B Quantification of inclusion levels of individual exons are shown as means with standard error from three experiments for embryos, larval brains, worker brains, drone brains and queen brains (*: $p<0.05$; **: $p<0.01$; ***: $p<0.001$; ****: $p<0.0001$).

Supplementary Figure 1

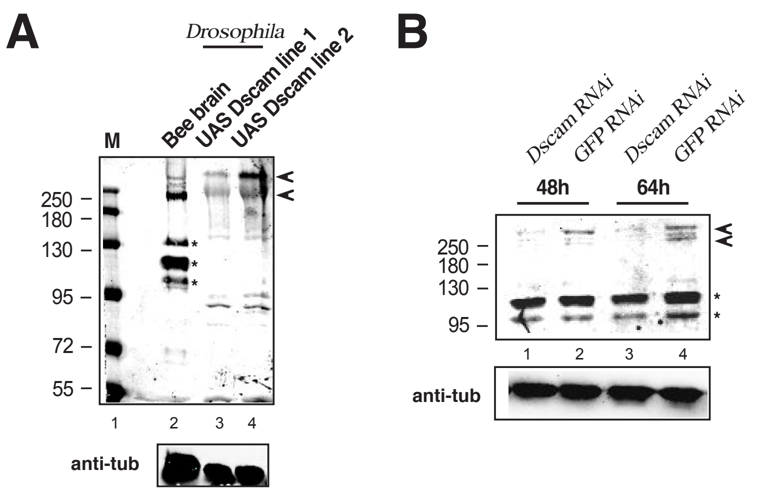

Supplementary Figure 2

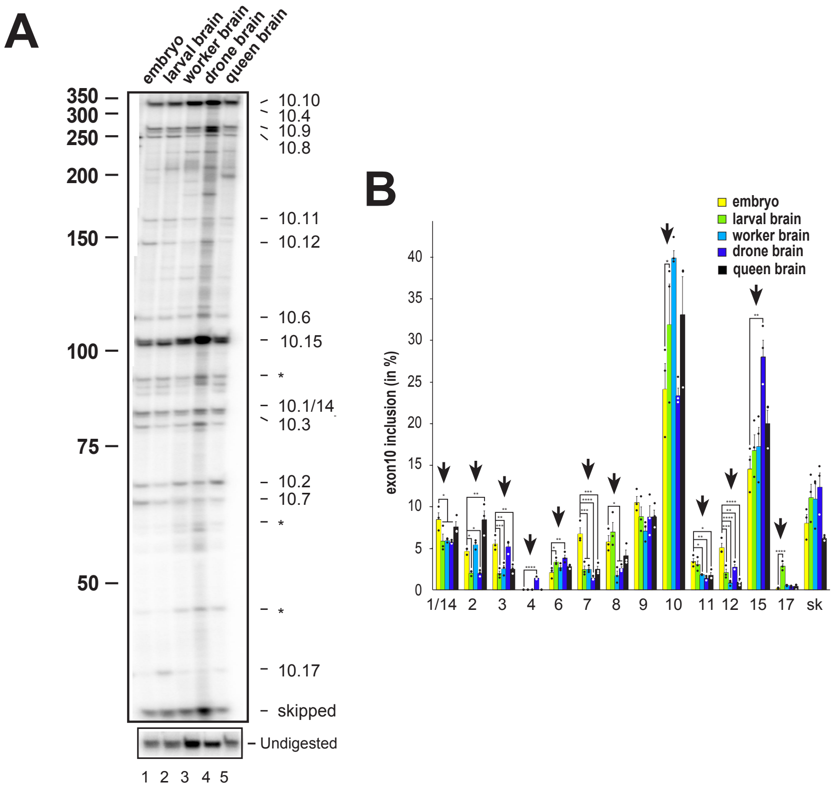

Supplement: Supplementary file 3 [file Data_Sheet_1.pdf]
